# Physiologically Based Pharmacokinetic Modeling for Mechanistic Analysis of Food Effects: A Case Study with Radotinib

**DOI:** 10.1007/s11095-026-04179-0

**Published:** 2026-08-21

**Authors:** Minseo Kang, Na Yun Kim, Jae Soo Shin, Min Soo Park, Eunjin Hong, Jangik I. Lee

**Affiliations:** 1 College of Pharmacy and Research Institute of Pharmaceutical Sciences, Seoul National University, Seoul, 08826 Republic of Korea; 2 IL-YANG Pharmaceutical Co., Ltd, Yongin, 17096 Republic of Korea; 3 Department of Clinical Pharmacology, Severance Hospital, Yonsei University College of Medicine, Seoul, 03722 Republic of Korea; 4 College of Pharmacy, Dongguk University, Goyang, 10326 Republic of Korea

**Keywords:** Clinical verification, Food effect, Physiologically based pharmacokinetic modeling, Radotinib, Sensitivity analysis

## Abstract

**Purpose:**

Physiologically based pharmacokinetic (PBPK) modeling has emerged as a promising tool for food effect assessment, yet its utility for poorly soluble and poorly permeable compounds has not been fully established. This study aimed to develop a PBPK model for radotinib, a second-generation tyrosine kinase inhibitor, to predict the food effect.

**Methods:**

The model was verified against plasma concentration–time (Cp-time) data obtained from a clinical trial conducted in healthy volunteers who received an oral dose of 400 mg under fasted and fed conditions. Sensitivity analyses were subsequently performed to identify the absorption-related parameters that influence the prediction of food effect.

**Results:**

All simulated-to-observed ratios for maximum Cp (C_max_), and area under the Cp-time curve from time zero to infinity (AUC_inf_) fell within two-fold acceptance criterion under fasted and fed conditions. Simulated geometric mean ratios for the food effect on C_max_ and AUC_inf_ were 2.65 and 3.47, which closely recapitulated the observed ratios of 2.65 and 3.49, respectively. Intrinsic solubility and the logarithm of the bile micelle-to-water partition coefficient for neutral species were identified as the primary determinants of food effect prediction.

**Conclusions:**

These findings demonstrate the utility of PBPK modeling for prediction of food effect on radotinib pharmacokinetics.

**Supplementary Information:**

The online version contains supplementary material available at https://doi.org/10.1007/s11095-026-04179-0.

## Introduction

The effect of food refers to the changes in the rate and extent of absorption of an orally administered drug under fed conditions as compared with those under fasted conditions. Such changes can result in clinically important differences in systemic exposure, which may subsequently affect therapeutic efficacy and safety. Approximately 40% of oral drugs approved by the Food and Drug Administration (FDA) or the European Medicines Agency (EMA) over the past decade were reported to exhibit food effects [1, 2]. Regulatory agencies therefore recommend submitting the results of food effect studies as part of new drug applications to support appropriate dosing guidance and product labeling [3, 4]. Particularly for poorly soluble and poorly permeable drugs, thorough characterization of food effects is critical. Postprandial increases in bile acid secretion enhance drug solubilization, while changes in gastric emptying and luminal pH exert opposing effects, which result in highly variable and unpredictable net food effects across compounds [5, 6].

Beagle dogs have traditionally been used in preclinical studies to predict food effect in humans, yet interspecies differences in gastrointestinal physiology compromise the translational reliability of this approach [7, 8]. Physiologically based pharmacokinetic (PBPK) modeling has emerged as a mechanistic alternative that incorporates human gastrointestinal physiology directly into the model structure. This integration of compound-specific properties such as solubility, permeability and bile micelle partitioning alongside physiological parameters governing luminal pH, bile secretion and gastric emptying enables prediction of food effects in humans without reliance on interspecies extrapolation [9–11]. Regulatory agencies have increasingly recognized PBPK modeling as a valuable tool for food effect prediction, with growing acceptance in regulatory submissions [12–14].

The prospective PBPK assessments for food effect have demonstrated variable predictive performance, with approximately 25% of predictions falling outside the two-fold acceptance criterion [9, 15], particularly for poorly soluble and poorly permeable compounds with complex absorption mechanisms [6]. For such compounds, the relative contributions of individual absorption-related parameters to PBPK-based predictive performance have not been fully characterized. One such compound is radotinib, a second-generation BCR-ABL tyrosine kinase inhibitor, which is characterized by moderate interindividual variability in systemic exposure, as well as an increased risk of dose-limiting toxicities at higher exposures [16–18]. As food-induced changes in the gastrointestinal environment may substantially alter radotinib exposure, understanding the influence of food on radotinib pharmacokinetics is essential for informing dosing strategy and minimizing the risk of dose-limiting toxicities.

Hence, the present study aimed to develop, for the first time, a PBPK model for radotinib and to evaluate the predictive performance of the model for the food effect against clinical data obtained from healthy volunteers (identifier, NCT06461078) [16]. A systematic sensitivity analysis was subsequently conducted to evaluate the impact of key absorption-related parameters on model performance, thereby assessing the robustness and mechanistic interpretability of the model. Collectively, the present study demonstrates the utility of PBPK modeling for food effect prediction and provides mechanistic insights into the food effect of radotinib.

## Methods

### PBPK Model Development

A PBPK model for radotinib was developed using Simcyp® Simulator (version 25; Certara, Sheffield, UK), with input parameters summarized in Table 1. The coefficients of variation for all input parameters were set at default values. Physicochemical parameters of radotinib including molecular weight, logarithm of acid dissociation constant (pK_a_) and logarithmic of the octanol–water partition coefficient (log P) were obtained from a public database [19] or calculated using Marvin® (ChemAxon, Budapest, Hungary). The fraction unbound in plasma (fu_p_) of radotinib was experimentally measured from a protein binding study conducted in human plasma. As the measured value was below 0.01, a value of 0.01 was used in accordance with ICH M12 guidance [20]. The blood-to-plasma concentration ratio (B/P) was predicted using the Simcyp internal predictor.

**Table 1:** Input parameters used for the physiologically based pharmacokinetic model (PBPK) of radotinib in Simcyp**®** version 25 (Certara)

Parameter	Value	Reference/Comments
**Physiochemical properties and blood binding**		
Molecular weight (g/mol)	530.5	[19]
Log P_o:w_	4.53	Calculated
Compound type	Diprotic base	
p*K*_a_ 1	6.48	Calculated
p*K*_a_ 2	12.73	
B/P	0.59	Predicted
fu_p_	0.01	In house data, [20]
**Absorption**		
Absorption model	ADAM model	
fu_gut_	1.0	Default
P_eff,man_ (× 10^−4^ cm/s)	0.57	Predicted
Caco-2 permeability (10^–6^ cm/sec)	1.52	In house data
Scalar	0.897	[21, 22]
Intrinsic solubility (mg/mL)	0.0013	Calculated
K_sp_	0.0001	Experimental (SIVA)
LogK_m:w, neutral_	6.764	Optimized (in house data)
LogK_m:w, ion_	4.764	
Formulation	Immediate release	
Lag time of fine particles, fasted (h)	0.14	In house data
Lag time of fine particles, fed (h)	1.30	[23]
**Distribution**		
Distribution model	Full PBPK model	
Prediction method	Method 1	[24]
V_ss_ (L/kg)	2.9836	In house data
K_p_ scalar	1.10	
**Elimination**		
CYP2C8		In house data
V_max_ (pmol/min/pmol)	0.05	
K_m_ (μM)	0.03	
CYP3A4		
V_max_ (pmol/min/pmol)	0.92	
K_m_ (μM)	0.42	
CYP3A5		
V_max_ (pmol/min/pmol)	0.97	
K_m_ (μM)	6.37	
fu_mic_	0.4	Predicted
ISEF	0.4	Calculated, [25–27]

*ADAM* advanced dissolution, absorption and metabolism, *B/P* blood-to-plasma ratio, *fu*_*gut*_ fraction unbound in the enterocyte, *fu*_*mic*_ fraction unbound in the *in vitro* microsomal incubation, *fu*_*p*_ fraction unbound in plasma, *ISEF* intersystem extrapolation factor, *K*_*m*_ Michaelis–Menten constant, *K*_*p*_ tissue-to-plasma partition coefficient, *K*_*sp*_ solubility product constant, *Log K*_*m:w*_ logarithmic micelle-to-water partition coefficient, *Log P*_*o:w*_ logarithmic partition coefficient octanol:water, *P*_*eff,man*_ effective permeability in man, *pK*_*a*_ logarithm of acid dissociation constant, *V*_*max*_ maximum rate of metabolism, *V*_*ss*_ volume of distribution at steady state

The absorption of radotinib was modeled using the Advanced Dissolution, Absorption and Metabolism (ADAM) model that incorporates mechanistic representations of gastrointestinal physiology. The effective permeability (P_eff_) of radotinib was estimated from Caco-2 cell permeability data (apical pH 6.5, basolateral pH 7.4) by applying a scalar correction factor of 0.897 to account for inter-laboratory variability based on comparison with the reported values of mannitol permeability under identical pH conditions [21, 22], yielding a final P_eff_ value of 0.57 × 10^–4^ cm/s. The intrinsic solubility (S_0_) of radotinib (free base) was estimated as 0.0013 mg/mL using the log S_0_ calculated by Marvin, which calculates intrinsic solubility from the molecular structure using a fragment-based method. The aqueous solubility of radotinib hydrochloride (salt) was separately measured across a pH range of 1.2 to 12 to derive a solubility product constant (K_sp_), where quantifiable solubility was obtained at pH 1.2 and pH 2.0 and fell below the lower limit of quantification at the higher pH values, which is consistent with the weakly basic nature of radotinib. Although weak base compounds are generally more soluble at lower pH, the measurement at pH 1.2 was excluded from the calculation because the presence of chloride ions in the pH 1.2 buffer suppresses the solubility of the chloride salt through a common ion effect. A solubility value of 0.00702 mg/mL measured at pH 2.0 was therefore used, from which K_sp_ of 0.0001 was calculated using the SIVA toolkit® (version 5; Certara, Sheffield, UK). K_sp_ together with the intrinsic solubility and pK_a_ was subsequently used to predict the pH-dependent solubility profile of the free base form.

In capturing food-mediated solubilization effects, bile micelle partitioning was incorporated via the logarithm of the bile micelle-to-water partition coefficient for neutral (log K_m:w,neutral_ = 6.764) and ionized (log K_m:w,ion_ = 4.764) species. Log K_m:w,neutral_ was optimized against internal plasma concentration–time (Cp-time) data obtained from a clinical trial of radotinib (identifier, NCT01602952; in house data), specifically those obtained following the first of repeated 400 mg doses under fasted conditions. Log K_m:w,ion_ was fixed at a value 2 log units below log K_m:w,neutral_, consistent with the Simcyp default relationship. Gastric emptying-related delays were accounted for by implementing a lag time for the appearance of fine particles in the intestine. Under fed conditions, the lag time was set based on literature-reported gastric emptying times for capsule formulations in the fed state [23], whereas the lag time was informed by in-house *in vitro* disintegration testing of radotinib (mean, 0.14 h; range, 0.10 h to 0.18 h) under fasted conditions. Apart from the lag time, all input parameters were identical under the fasted and fed conditions.

The distribution of radotinib was modeled using the full PBPK framework. The volume of distribution at steady state (V_ss_) in humans was predicted to be 2.9836 L/kg using the corrected method of Poulin and Theil (Method 1) [24]. The scalar of 1.10 for tissue-to-plasma partition coefficient (Kp) was derived from the parameters obtained from pharmacokinetic studies in rats, using the Simcyp internal predictor based on the corrected method of Poulin and Theil (Method 1) [24]. The parameters included the apparent V_ss_ (V_ss,rat_/F_rat_), oral bioavailability (F_rat_) and fu_p_ of radotinib obtained in rat studies (fu_p,rat_), with values of 5.25 L/kg, 0.65 and 0.01, respectively.

The hepatic elimination of radotinib was computed using Michaelis constant (K_m_) and maximum reaction velocity (V_max_) obtained from *in vitro* assays with recombinant cytochrome P450 enzymes (rhCYP), specifically CYP2C8, CYP3A4 and CYP3A5. The fraction unbound in microsomes (fu_mic_) of 0.4 was predicted using the Simcyp internal predictor based on log P and the protein concentration of microsomal incubation. An intersystem extrapolation factor (ISEF) of 0.4 was calculated by scaling V_max_ from recombinant CYP assays to human liver microsomes (HLM) based on the abundance of each CYP enzyme in HLM [25–27], using the following equation:

$$ISEF\;\left({fromV}_{max}\right)=\frac{V_{max_{ij}}\left(HLM\right)}{\sum_{j=1}^n\sum_{i=1}^nV_{{max}_i\;}\left({rhCYP}_j\right)\times{CYP}_j\;abundance\;\left(HLM\right)}$$

Based on these parameters, the metabolism of radotinib was predicted to be mediated predominantly by CYP3A4, with mean fractions metabolized (fm) of approximately 88% for CYP3A4, 11% for CYP2C8, and 1% for CYP3A5.

### PBPK Model Verification of Radotinib

The PBPK model was verified against Cp-time data obtained from a clinical trial performed in 24 Chinese patients with chronic myeloid leukemia (CML) [16], in which radotinib was administered repeatedly at 300 mg twice daily under fasted conditions (identifier, NCT03722420). Simulations were performed over 14 days following the first administration using the default Sim-Chinese Healthy Volunteers population library in Simcyp to match the ethnicity of the clinical trial. Each simulation consisted of 24 subjects across 10 trials, with an age range of 21 to 65 years and a female proportion of 0.46 to match the design of the study.

Following verification under fasted condition, the model was further evaluated for its ability to reproduce the observed food effect using Cp-time data obtained from a clinical trial of radotinib conducted in 24 healthy male volunteers [16]. In this study, a single oral dose of 400 mg was administered under fasted and fed conditions (identifier, NCT06461078), an independent dataset distinct from that used for parameter optimization. Simulations under both conditions were performed using the default Sim-Healthy Volunteers population library in Simcyp over a 72-h sampling period. Each simulation comprised 24 subjects across 10 trials, with an age range of 20 to 51 years and a female proportion of 0 to match the design of the clinical trial. Consistent with the clinical trial, fasted subjects were assumed to be in an overnight fasted state, while fed subjects were assumed to receive a meal of 800 kcal and 30% fat, with radotinib administered 30 min after the start of the meal.

The predictive performance of the PBPK model was assessed by calculating the ratios of means of simulated to observed values for maximum Cp (C_max_) and area under the Cp-time curve (AUC) from time zero to 12 h (AUC_0–12 h_) in patients with CML, reflecting the 12-h sampling interval of the twice-daily regimen. In healthy volunteers, the corresponding ratios were calculated for C_max_, AUC from time zero to 72 h (AUC_0–72 h_) and to infinity (AUC_inf_). The ability to predict the food effect was assessed by comparing the simulated geometric mean ratios (GMRs) of C_max_, AUC_0–72 h_ and AUC_inf_ between fed and fasted conditions with those observed in the clinical trial [16]. Model performance was considered adequate if simulated-to-observed ratios fell within a two-fold range, consistent with commonly applied criteria for PBPK model evaluation in the literature [9, 15].

### Sensitivity Analysis

A sensitivity analysis was performed to evaluate the impact of key model parameters on the prediction of food effects on radotinib pharmacokinetics. As food effect on drug exposure is primarily mediated through changes in gastrointestinal absorption processes [2], the analysis was restricted to absorption-related parameters (i.e., intrinsic solubility, P_eff_, log K_m:w,neutral_, log K_m:w,ion_ and lag time of fine particles under fed conditions), with systemic disposition parameters held unchanged.

A local sensitivity analysis was first performed by varying each parameter individually. Intrinsic solubility was explored across a tenfold range, encompassing the reported prediction error for in silico aqueous solubility estimation of crystalline compounds [28, 29]. P_eff_ was likewise explored across a tenfold range, while log K_m:w,neutral_ and log K_m:w,ion_ ranged from −1 to + 1 log unit, corresponding to an equivalent tenfold range. The lag time under fed conditions was varied from 0.25 h to 3 h based on reported ranges of gastrointestinal transit in the fed state [23, 30, 31]. At each parameter value, the simulated GMR for C_max_ and AUC_0–72 h_ was compared against the simulated baseline value to assess the influence of each parameter on the food effect. The impact of each parameter on time to C_max_ (T_max_) was additionally assessed.

A subsequent global sensitivity analysis was performed in two stages. First, a Morris elementary-effects screening was conducted to evaluate the influence of intrinsic solubility, P_eff_, log K_m:w,neutral_, and log K_m:w,ion_ on the food effect [32, 33], using four levels and one hundred trajectories across the same ranges as in the local analysis for the fasted and fed states. Second, a joint perturbation analysis was performed for the parameters that affected the food effect, which were varied together across their ranges in all combinations. For each combination, the simulated GMR for C_max_ and AUC_0–72 h_ was compared against the simulated baseline value.

## Results

### Performance of Radotinib PBPK Model

#### Prediction of Pharmacokinetic Parameters Under Fasted and Fed Conditions

The PBPK model also effectively reproduced the observed mean Cp-time profiles in patients with CML following repeated doses of radotinib 300 mg twice daily under fasted conditions, with most observed concentrations falling within the simulated 5th to 95th percentile range on Day 1 and all falling within the range on Day 14 (Supplementary Fig. 1). The mean values of simulated T_max_ closely agreed with the observed values on Day 1 (3.1 h *versus* 3.2 h) and Day 14 (2.7 h *versus* 2.8 h). On Day 1, the simulated values of C_max_ and AUC_0–12 h_ (477 ng/mL and 4,039 ng·h/mL) closely matched the observed values (587 ng/mL and 3,875 ng·h/mL), with the simulated-to-observed ratios of 0.81 and 1.04, respectively (Table 2). On Day 14, the simulated C_max_ and AUC_0–12 h_ (1,705 ng/mL and 17,914 ng·h/mL) were consistent with the observed values (1,780 ng/mL and 15,290 ng·h/mL), which yielded simulated-to-observed ratios of 0.96 and 1.17, respectively (Table 2). All simulated-to-observed ratios fell within the pre-specified two-fold acceptance criterion, which demonstrates adequate predictive performance of the PBPK model under repeated-dose conditions in patients with CML.

**Table 2:** Comparison of simulated and observed pharmacokinetic parameters for the verification of physiologically based pharmacokinetic model for radotinib (patients with chronic myeloid leukemia, fasted conditions)

	Simulated			Observed		
	T_max_ (h)	C_max_ (ng/mL)	AUC_0–12 h_ (ng∙h/mL)	T_max_ (h)	C_max_ (ng/mL)	AUC_0–12 h_ (ng∙h/mL)
**Day 1**						
**Mean**	3.1	477	4039	3.2	587	3875
**CV (%)**	44.3	38.7	40.8	34.1	62.2	64.6
**Simulated/Observed**	0.97	0.81	1.04			
**Day 14**						
**Mean**	2.7	1705	17,914	2.8	1780	15,290
**CV (%)**	49.0	74.4	85.7	56.4	44.4	42.9
**Simulated/Observed**	0.96	0.96	1.17			

*AUC*_*0–12 h*_ area under the plasma concentration–time curve from time zero to 12 h, *AUC*_*inf*_ area under the plasma concentration–time curve from time zero to infinity, *C*_*max*_ maximum plasma concentration, *CV* coefficient of variance, *T*_*max*_ time to reach maximum plasma concentration

**Fig. 1:**
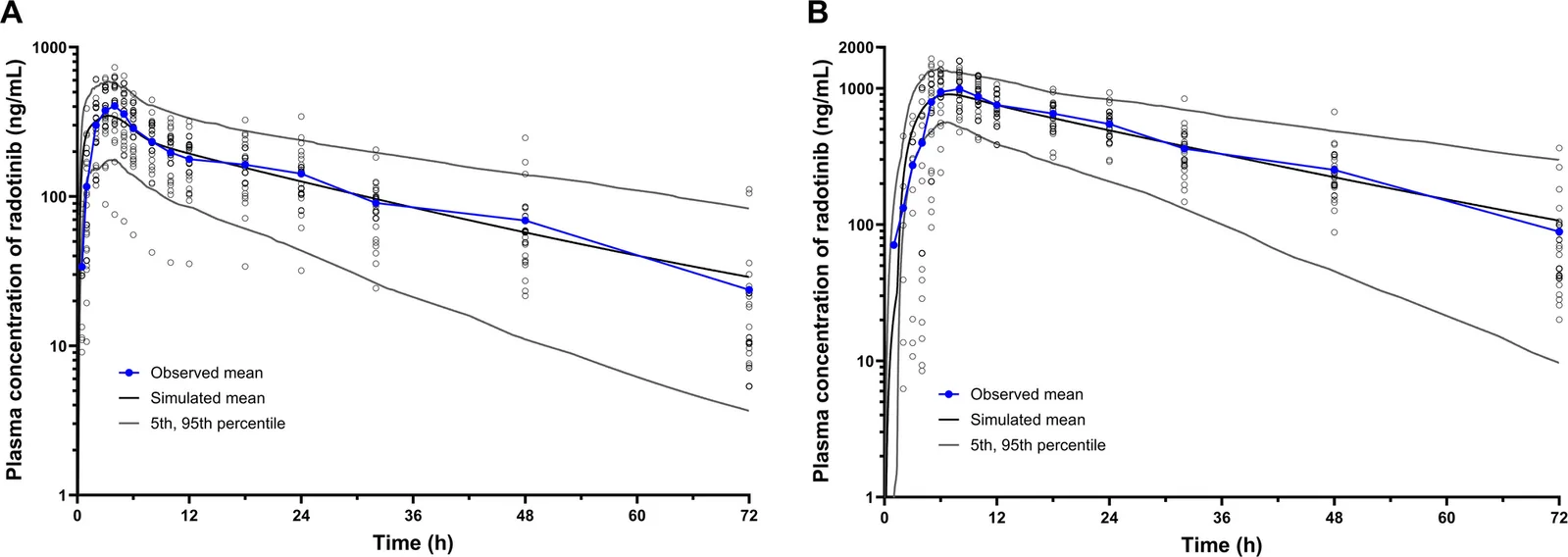
Simulated and observed plasma concentration–time profiles of radotinib under (**A**) fasted and (**B**) fed conditions. Circles stand for observed concentrations, blue line for observed mean, black line for simulated mean, and gray lines for the 5th and 95th percentiles of simulated concentrations.

The PBPK model also effectively captured the observed mean Cp-time profiles in healthy volunteers following a single oral dose of 400 mg under fasted and fed conditions, with the observed concentrations largely falling within the simulated 5th to 95th percentile range (Fig. 1). The mean values of simulated T_max_ were consistent with the observed values under both fasted (3.2 h *versus* 3.4 h) and fed conditions (7.2 h *versus* 7.2 h). Under fasted conditions, the simulated values of C_max_, AUC_0–72 h_ and AUC_inf_ (367 ng/mL, 8,053 ng·h/mL and 9,236 ng·h/mL) were in agreement with the observed values (458 ng/mL, 8,076 ng·h/mL and 9,196 ng·h/mL), with the simulated-to-observed ratios of 0.80, 1.00, and 1.00, respectively (Table 3). Under fed conditions, the simulated C_max_, AUC_0–72 h_ and AUC_inf_ (947 ng/mL, 26,996 ng·h/mL and 31,135 ng·h/mL) were consistent with observed values (1,196 ng/mL, 27,099 ng·h/mL and 30,537 ng·h/mL), which resulted in simulated-to-observed ratios of 0.79, 1.00, and 1.02, respectively (Table 3). All simulated-to-observed ratios fell within the pre-specified two-fold acceptance criterion, which demonstrates adequate predictive performance of the PBPK model under both fasted and fed conditions. Apart from the ratio of C_max_ under fed conditions, all other ratios even met the more stringent bioequivalent range of 0.80 to 1.25.

**Table 3:** Comparison of simulated and observed pharmacokinetic parameters for the verification of physiologically based pharmacokinetic model for radotinib (healthy volunteers, fasted and fed conditions)

	Simulated				Observed			
	T_max_ (h)	C_max_ (ng/mL)	AUC_0-72h_ (ng∙h/mL)	AUC_inf_ (ng∙h/mL)	T_max_ (h)	C_max_ (ng/mL)	AUC_0-72h_ (ng∙h/mL)	AUC_inf_ (ng∙h/mL)
**Fasted**								
**Mean**	3.2	367	8053	9236	3.4	458	8076	9196
**CV (%)**	33.8	35.9	49.7	58.6	27.9	32.6	43.9	57.9
**Simulated/Observed**	0.94	0.80	1.00	1.00				
**Fed**								
**Mean**	7.2	947	26,966	31,135	7.2	1196	27,099	30,537
**CV (%)**	22.7	26.8	40.1	51.9	41.3	24.1	27.6	36.5
**Simulated/Observed**	1.00	0.79	1.00	1.02				

*AUC*_*0–72 h*_ area under the plasma concentration–time curve from time zero to 72 h, *AUC*_*inf*_ area under the plasma concentration–time curve from time zero to infinity, *C*_*max*_ maximum plasma concentration, *CV* coefficient of variance, *T*_*max*_ time to reach maximum plasma concentration

#### Prediction of Food Effect

The PBPK model reliably predicted the food effect on radotinib pharmacokinetics, with simulated GMRs of 2.65, 3.47, and 3.47 for C_max_, AUC_0–72 h_ and AUC_inf_, respectively, which closely recapitulated the observed GMRs of 2.65, 3.49, and 3.49 (Table 4). All simulated GMRs fell within the pre-specified two-fold acceptance criterion, which supports the capacity of the developed PBPK model to quantitatively predict the food effect.

**Table 4:** Comparison of the simulated and observed food effect on radotinib pharmacokinetics

	Simulated			Observed		
	C_max_	AUC_0-72h_	AUC_inf_	C_max_	AUC_0-72h_	AUC_inf_
GMR^*^	2.65	3.47	3.47	2.65 (2.27–3.10)	3.49 (2.98–4.08)	3.49 (2.94–4.14)
**Simulated/Observed**	1.00	0.99	0.99			

*AUC*_*0–72 h*_ area under the plasma concentration–time curve from time zero to 72 h, *AUC*_*inf*_ area under the plasma concentration–time curve from time zero to infinity, *C*_*max*_ maximum plasma concentration, *GMR* geometric mean ratio

^*^Observed values are presented as the geometric mean ratio with the 90% confidence interval in parentheses. Simulated values are presented as the geometric mean ratio of the virtual population

### Sensitivity Analysis

The influence of each absorption-related parameter on the food effect was first evaluated individually by local sensitivity analysis (Sects. Intrinsic Solubility, Effective Permeability, Bile Micelle-to-Water Partition Coefficient, Lag Time of Fine Particles Under Fed Conditions), followed by a global sensitivity analysis (Sect. "Global Sensitivity Analysis").

#### Intrinsic Solubility

**Fig. 2:**
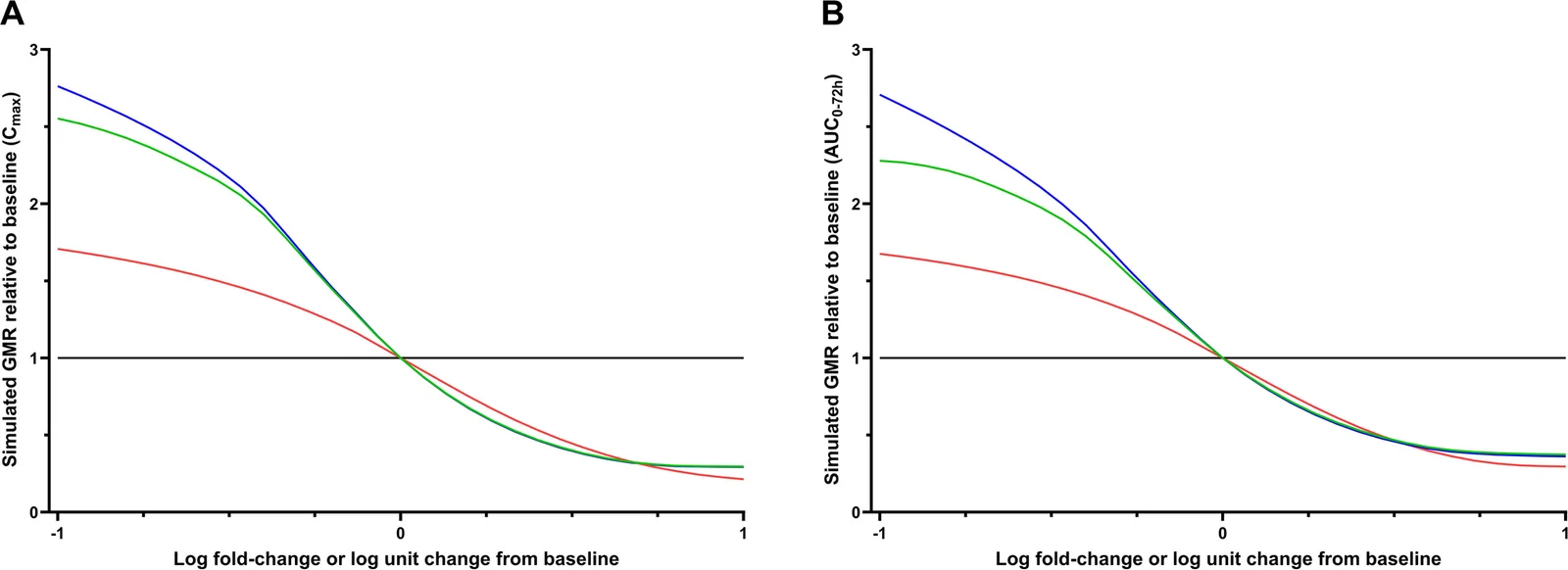
Sensitivity of the simulated fed-to-fasted geometric mean ratio (GMR) to changes in absorption-related parameters for (**A**) maximum plasma concentration (C_max_) and (**B**) area under the plasma concentration–time curve from time zero to 72 h (AUC_0–72 h_). The simulated GMR relative to the simulated baseline GMR is shown as a function of parameter variation for intrinsic solubility (blue), effective permeability (red), logarithm of the bile micelle-to-water partition coefficient for the neutral species (log K_m:w,neutral_; green), and logarithm of the bile micelle-to-water partition coefficient for the ionized species (log K_m:w,ion_; black). For intrinsic solubility and effective permeability, the x-axis represents the log fold-change from the baseline value. For log K_m:w,neutral_ and log K_m:w,ion_, the x-axis represents the log unit change from the baseline value.

Intrinsic solubility, with a baseline value of 0.0013 mg/mL in the PBPK model for radotinib, was varied across a tenfold range from 0.00013 mg/mL to 0.013 mg/mL. Lower intrinsic solubility substantially decreased the simulated C_max_ and AUC_0–72 h_ under fasted conditions while the corresponding decreases under fed conditions were less pronounced, which resulted in overprediction of the food effect on radotinib pharmacokinetics (Fig. 2, Supplementary Fig. 2). At the lowest value of intrinsic solubility examined, the simulated GMR for C_max_ and AUC_0–72 h_ were increased by 2.76-fold and 2.71-fold relative to their simulated baseline values, respectively. Higher intrinsic solubility increased the simulated C_max_ and AUC_0–72 h_ under fasted conditions while the corresponding values under fed conditions showed minimal change, which led to underprediction of the food effect. At the highest value examined, the simulated GMR for C_max_ and AUC_0–72 h_ were decreased by 71% and 64% relative to their simulated baseline values, respectively. These findings suggest that for the drugs with low solubility and low permeability, higher intrinsic solubility may increase fasted-state absorption to a greater extent than fed-state absorption, thereby potentially attenuating the food effect.

With respect to T_max_, the simulated T_max_ under fasted conditions was increased by 49% to 4.77 h at the lowest value examined and decreased by 39% to 1.96 h at the highest value examined. The simulated T_max_ under fed conditions was increased by 70% to 10.22 h at the lowest value examined and decreased by 4% to 5.77 h at the highest value examined. Overall, intrinsic solubility markedly influenced T_max_, with reduced solubility prolonging absorption, particularly under fed conditions, whereas increased solubility accelerated absorption predominantly in the fasted state, indicating that solubility-driven dissolution kinetics play a key role in governing the rate of absorption and its differential modulation by food.

#### Effective Permeability

P_eff_, with a baseline value of 0.57 × 10^–4^ cm/s in the PBPK model, was varied across a tenfold range from 0.057 × 10^–4^ cm/s to 5.70 × 10^–4^ cm/s. Lower P_eff_ decreased the systemic exposure of radotinib under fasted and fed conditions to a relatively comparable extent, which resulted in a less pronounced overprediction of the food effect relative to intrinsic solubility (Fig. 2, Supplementary Fig. 3). At the lowest value examined, the simulated GMR for C_max_ and AUC_0–72 h_ were increased by 1.71-fold and 1.67-fold relative to their simulated baseline values, respectively. Higher P_eff_ progressively augmented the simulated C_max_ and AUC_0–72 h_ under fasted conditions while the corresponding values under fed conditions exhibited negligible change, which resulted in underprediction of the food effect. At the highest value examined, the simulated GMR for C_max_ and AUC_0–72 h_ were decreased by 79% and 70% relative to their simulated baseline values, respectively. These findings suggest that, for radotinib, the food effect is less sensitive to changes in P_eff_ than to changes in intrinsic solubility. With respect to T_max_, the simulated T_max_ under fasted conditions was increased by 11% to 3.54 h at the lowest value examined and decreased by 47% to 1.70 h at the highest value examined. The simulated T_max_ under fed conditions was increased by 22% to 7.36 h at the lowest value examined and decreased by 46% to 3.24 h at the highest value examined.

#### Bile Micelle-to-Water Partition Coefficient

Log K_m:w,neutral_, with a baseline value of 6.764 in the PBPK model, was varied across a ± 1 log unit range from 5.764 to 7.764. Lower log K_m:w,neutral_ markedly decreased the simulated C_max_ and AUC_0–72 h_ under fasted conditions while the corresponding decreases under fed conditions were more attenuated, which yielded overprediction of the food effect (Fig. 2, Supplementary Fig. 4). At the lowest value examined, the simulated GMR for C_max_ and AUC_0–72 h_ were increased by 2.55-fold and 2.28-fold relative to their simulated baseline values, respectively. Higher log K_m:w,neutral_ enhanced the simulated C_max_ and AUC_0–72 h_ under fasted conditions while the corresponding values under fed conditions displayed marginal change, which resulted in underprediction of the food effect. At the highest value examined, the simulated GMR for C_max_ and AUC_0–72 h_ were decreased by 70% and 63% relative to their simulated baseline values, respectively. With respect to T_max_, the simulated T_max_ under fasted conditions was increased by 52% to 4.86 h at the lowest value examined and decreased by 40% to 1.92 h at the highest value examined. The simulated T_max_ under fed conditions was increased by 70% to 10.21 h at the lowest value examined and decreased by 4% to 5.77 h at the highest value examined. These findings suggest that for drugs with low solubility and low permeability, the degree of bile micelle partitioning will likely influence the magnitude of the food effect as well as absorption kinetics.

Log K_m:w,ion_, with a baseline value of 4.764 in the PBPK model, was varied across a ± 1 log unit range from 3.764 to 5.764. In contrast to log K_m:w,neutral_, log K_m:w,ion_ had negligible impact on the simulated food effect across the range examined, with the simulated C_max_ and AUC_0–72 h_ under both fasted and fed conditions showing similarly marginal changes throughout. The simulated T_max_ also remained unchanged at approximately 3.19 h under fasted conditions and 6.01 h under fed conditions across the full range examined. These findings suggest that bile micelle-mediated solubilization of radotinib is driven primarily by the neutral rather than the ionized species.

#### Lag Time of Fine Particles Under Fed Conditions

**Fig. 3:**
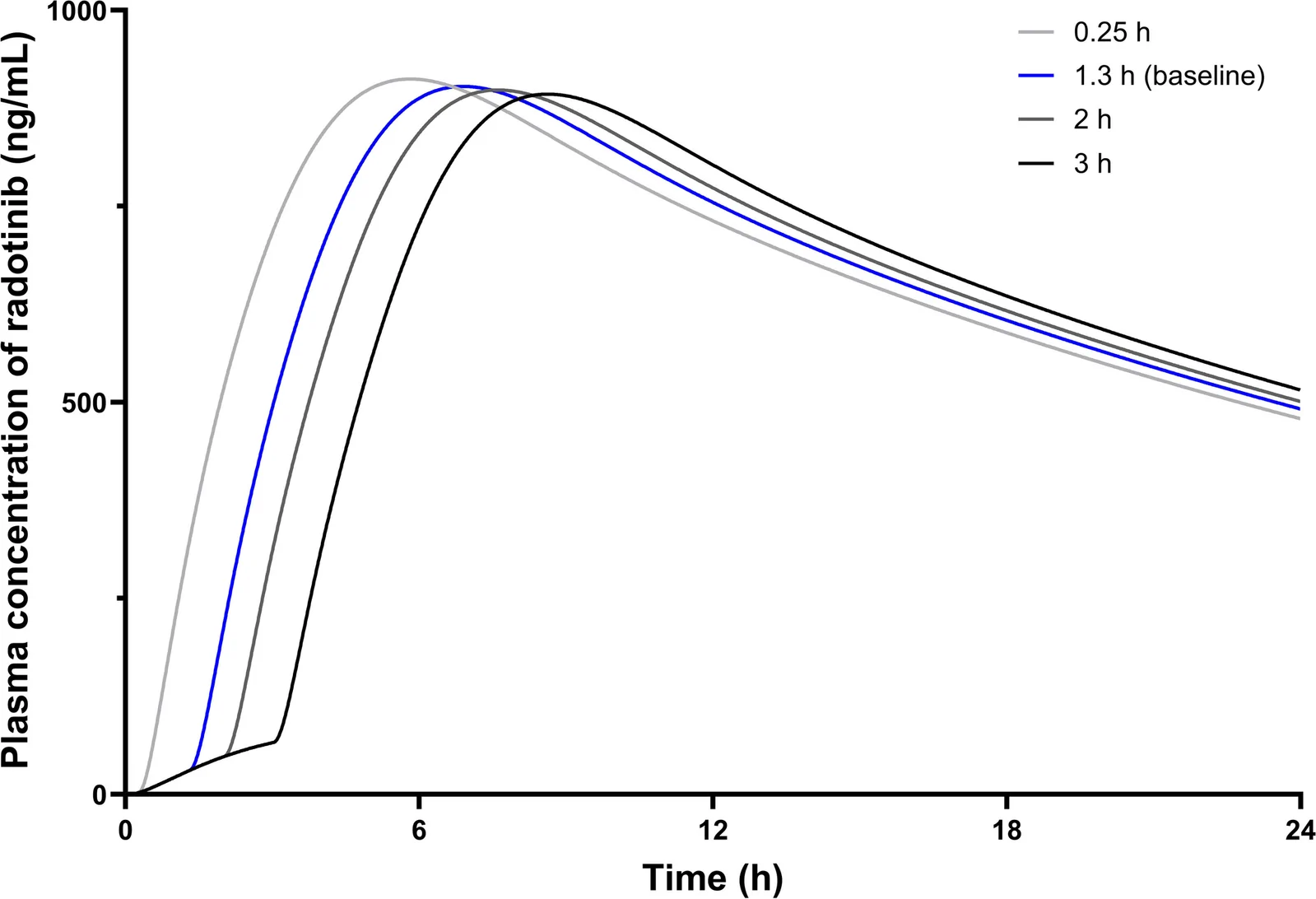
Simulated mean plasma concentration–time profiles of radotinib under fed conditions at lag times of fine particles of 0.25 h (light gray), 1.3 h (blue, baseline), 2 h (dark gray), and 3 h (black).

The lag time of fine particles under fed conditions, with a baseline value of 1.3 h in the PBPK model, was examined across a range from 0.25 h to 3.0 h. Variations of the lag time across this range did not meaningfully affect the simulated food effect, with the simulated GMR for C_max_ and AUC_0–72 h_ changing by less than 2% of their simulated baseline values. In contrast, lag time exerted a pronounced effect on absorption kinetics, as reflected by the simulated T_max_ under fed conditions, which decreased by 18% to 4.91 h at the lowest value examined and increased by 29% to 7.74 h at the highest value examined (Fig. 3).

#### Global Sensitivity Analysis

Morris screening was performed to further evaluate the influence of absorption-related parameters on radotinib exposure. Consistent with the local sensitivity analysis, intrinsic solubility, P_eff_, and log K_m:w,neutral_ were identified as influential across the evaluated ranges, whereas log K_m:w,ion_ showed negligible effects. The ratio of the standard deviation of the elementary effects (σ) to the mean absolute elementary effect (μ^*^) was used to assess nonlinearity or interaction in parameter effects. For intrinsic solubility and log K_m:w,neutral_, the σ/μ^*^ ratios for AUC_0–72 h_ were 0.95 and 0.89 under fasted conditions, and 1.19 and 1.01 under fed conditions, respectively, suggesting nonlinear or interactive effects on radotinib exposure. In contrast, P_eff_ showed lower σ/μ^*^ ratios of 0.69 and 0.37 under fasted and fed conditions, respectively, indicating a comparatively more monotonic influence.

Based on these findings, intrinsic solubility, P_eff_, and log K_m:w,neutral_ were included in the joint perturbation analysis to evaluate their combined and potentially interactive effects on the simulated GMRs for C_max_ and AUC_0–72 h_. Log K_m:w,ion_ was excluded because it showed negligible influence on the simulated GMRs in both the local and Morris analyses. The lag time under fed conditions was also excluded because the local analysis showed that it primarily affected T_max_ under fed conditions, with limited impact on the simulated GMRs.

In the joint analysis, combined parameter uncertainty is summarized in Table SI as the fold-changes in the simulated GMRs for C_max_ and AUC_0–72 h_ relative to the baseline simulation. Across the evaluated combinations, the GMR for AUC_0–72 h_ varied from 0.29- to 2.88-fold and the GMR for C_max_ varied from 0.15- to 2.84-fold relative to baseline (Table SI). The GMR for AUC_0–72 h_ was highest when P_eff_ and intrinsic solubility were decreased, whereas the GMR for C_max_ was highest when intrinsic solubility and log K_m:w,neutral_ were decreased. The lowest GMRs for both endpoints occurred when P_eff_ and intrinsic solubility were increased together. In contrast, opposite directional changes in intrinsic solubility and log K_m:w,neutral_ kept the simulated GMRs within approximately 1.0- to 1.1-fold of baseline. Together, these findings indicate that the simulated GMRs were influenced not only by individual parameter changes but also by the direction and combination of changes in solubility-related and permeability parameters.

## Discussion

In this study, the PBPK model for radotinib was developed to quantitatively predict the food effect on systemic exposure and to identify the key absorption-related parameters that influence the food effect. This retrospective analysis integrated *in vitro* and in silico data to elucidate the mechanistic basis of the observed food effect on radotinib pharmacokinetics. The model reasonably described the pharmacokinetic characteristics of radotinib under both fasted and fed conditions, with all simulated-to-observed ratios falling within the two-fold range (Tables 2 and 3). Of importance, the model adequately predicted the magnitude of the food effect, with simulated GMRs for C_max_, AUC_0–72 h_ and AUC_inf_ consistent with the observed values (Table 4). These findings support the utility of PBPK modeling as a mechanistic approach to predict clinically relevant food effects.

Mechanistically, the sensitivity analysis provides an important insight into the drivers of the food effect for radotinib. Variations in intrinsic solubility and log K_m:w,neutral_ had a substantially greater impact on simulated exposure to radotinib under fasted conditions than under fed conditions (Fig. 2, Supplementary Figs. 2 and 4). Under fasted conditions, since the concentrations of bile micelles in the intestinal lumen are low [34, 35], the exposure to radotinib appears to be sensitive to changes in intrinsic solubility. The sensitivity of the food effect prediction to log K_m:w,neutral_, which is comparable with that observed for intrinsic solubility, suggests that even the small quantities of bile micelles presented under fasted conditions contribute meaningfully to the solubilization of radotinib, likely because of the particularly low aqueous solubility of radotinib. In contrast, under fed conditions, the postprandial increase in bile salt and lecithin concentrations substantially enhances bile micelle-mediated solubilization [35, 36], which made the exposure to radotinib less sensitive to reductions in intrinsic solubility or log K_m:w,neutral_ than under fasted conditions. Increases in these parameters led to a plateau in the exposure under fed conditions, as the fraction absorbed (fa) had approached saturation (0.89 for intrinsic solubility and 0.84 for log K_m:w,neutral_). These findings indicate that the food effect observed for low-solubility drugs, such as radotinib, is primarily driven by solubility-limited absorption in the fasted state, with bile micelle-mediated solubilization playing a key modulatory role.

In contrast to solubility-related parameters, reductions in P_eff_ decreased the simulated exposure to radotinib under both fasted and fed conditions to a similar extent (Fig. 2, Supplementary Fig. 3), resulting in minimal impact on the predicted food effect. The prediction of food effect consequently fell within the two-fold acceptance criterion across the full range of reductions examined. At one-tenth of the baseline value of P_eff_, the simulated GMRs for C_max_ and AUC_0–72 h_ increased by 1.71-fold and 1.67-fold relative to their simulated baseline values, respectively. This pattern indicates that, while permeability constrains the absorption of radotinib [37, 38], that does not drive the differential exposure between fed and fasted states. Increases in P_eff_ similarly led to a plateau in the exposure under fed conditions, as the fa reached saturation (fa = 1.00), while the exposure under fasted conditions continued to rise, a pattern consistent with that observed for solubility-related parameters. Collectively, these findings indicate that solubility-limited absorption in the fasted state is the primary driver of the food effect for radotinib, with permeability-limited absorption playing a secondary role, consistent with previous reports for poorly soluble compounds [37, 39, 40].

The local and global sensitivity analyses together indicate that the predicted food effect of radotinib reflects the combined contribution of absorption-related processes, rather than the effect of any single parameter alone. In the joint perturbation analysis, opposite directional changes in intrinsic solubility and log K_m:w,neutral_ kept the simulated GMRs close to the baseline values, within approximately 1.0- to 1.1-fold of baseline (Table SI). This finding suggests that intrinsic solubility and bile micelle partitioning should be interpreted as jointly contributing to the effective solubilization of radotinib in the intestinal lumen, rather than as fully independent determinants of the food-effect prediction. Reductions in solubilization-related capacity, in turn, increased the simulated GMRs, supporting the interpretation that the magnitude of the food effect becomes amplified when absorption under fasted conditions is more strongly solubility-limited. Together, these findings refine the local sensitivity-based interpretation by showing that the magnitude of the predicted food effect is affected by combined parameter uncertainty, while the overall solubilization-driven interpretation remains consistent with the local sensitivity results.

The simulated T_max_ was most sensitive to changes in intrinsic solubility and log K_m:w,neutral_ under both fasted and fed conditions, with the influence of P_eff_ being less pronounced. This pattern suggests that solubility-related parameters and permeability will likely serve as primary and secondary contributors, respectively, to the rate of radotinib absorption, consistent with their established roles in determining the extent of absorption. The lag time of fine particles under fed conditions appears to selectively influence the simulated T_max_ under fed conditions, with longer lag times associated with a progressive delay in the onset of radotinib absorption, consistent with prolonged gastric residence time and delayed intestinal release of drug particles in the fed state [8, 23].

Of interest, the food effect on radotinib shares similar mechanistic features with those reported for poorly soluble, highly permeable compounds such as efavirenz and cinnarizine, in which solubility-limited absorption in fasted conditions has been identified as the primary driver of positive food effect [34]. For efavirenz and cinnarizine, fa under fed conditions approached near-complete absorption (fa = 1.00 and 0.99, respectively), whereas the simulated fa for radotinib reached a plateau of 0.89 and 0.84 in the sensitivity analyses for intrinsic solubility and log K_m:w,neutral_, respectively. This suggests that the presence of a permeability barrier may attenuate the increase in systemic exposure afforded by food-induced solubilization even when solubility constitutes the dominant rate-limiting step for absorption, which implies that PBPK modeling is well suited for characterizing the food effect mechanistically.

This study has some limitations that warrant consideration. The intrinsic solubility of radotinib was estimated using a computational tool, as measured solubility values fell below the lower limit of quantification at higher pH values, consistent with the weakly basic nature of radotinib. The uncertainty associated with this estimate might be partially mitigated by anchoring the pH-dependent solubility profile to a K_sp_ derived from experimental measurements. Uncertainty also remained in the bile micelle partitioning parameter, as direct *in vitro* bile micelle partitioning data were not available for radotinib. Although biorelevant solubility was evaluated, the solubility in FaSSIF was below the lower limit of quantification. Given this limitation, log K_m:w,neutral_ could not be reliably estimated from biorelevant solubility measurements and was instead optimized using an independent clinical fasted-state concentration–time dataset. Accordingly, the mechanistic interpretation of intrinsic solubility and log K_m:w,neutral_ should be made cautiously. The remaining uncertainty associated with these parameters was evaluated through the local sensitivity and joint analyses, the results of which should be considered when interpreting the simulated GMRs*.* In addition, particle size distribution, supersaturation, and precipitation of radotinib were not characterized experimentally and were therefore represented by default model parameters, although these processes are known to influence the rate and extent of oral absorption of poorly soluble compounds [41]. Moreover, the PBPK model was verified against oral Cp-time data only and is best regarded as an absorption-focused model. Further verification with intravenous or multiple dose-level data would be required for it to be considered a fully qualified systemic PBPK model. In addition, because of the limited availability of published pharmacokinetic data, the model could not be verified at dose levels higher than 400 mg or under alternative formulations. Such verification would further strengthen the model. The model also underpredicted C_max_ under both fasted and fed conditions to a comparable extent, so the close agreement between the simulated and observed GMRs of C_max_ should not be overinterpreted, given that this comparable bias partly cancels in the ratio. The prediction of the food effect, however, relies primarily on AUC, which the model predicted without comparable bias under both conditions. This prediction therefore remains robust despite the underprediction of absolute C_max_.

Despite these limitations, the model adequately described the pharmacokinetics of radotinib under both fasted and fed conditions, and reproduced the magnitude of the food effect, supporting the robustness of the primary outcomes of this study. The present study represents the first mechanistic characterization of the food effect for radotinib using a PBPK approach that incorporates human gastrointestinal physiology. These findings support the utility of PBPK modeling for assessing the food effects of compounds with complex absorption characteristics.

## Conclusion

For the first time, the PBPK model for radotinib was developed and verified in this study. The PBPK model adequately predicted the food effect on radotinib pharmacokinetics, with the simulated geometric mean ratios closely recapitulating the observed ratios within the two-fold acceptance criterion. Sensitivity analysis identified intrinsic solubility and bile micelle-to-water partition coefficient for neutral species as the primary determinants of the food effect. This indicates that solubility-limited absorption in the fasted state mainly drives the food effect of radotinib, a poorly soluble compound. These findings support the utility of PBPK modeling in predicting food effects for poorly soluble compounds.

## Supplementary Information

Below is the link to the electronic supplementary material.

**Supplementary Figure 1** Simulated and observed plasma concentration-time profiles of radotinib on (A) Day 1 and (B) Day 14 in patients with chronic myeloid leukemia under fasted conditions. Circles stand for observed concentrations, blue line for observed mean, black line for simulated mean, and gray lines for the 5th and 95th percentiles of simulated concentrations. (JPG 1797 KB)

**Supplementary Figure 2** Sensitivity of the simulated (A) maximum plasma concentration (C_max_) and (B) area under the plasma concentration-time curve from time zero to 72 hours (AUC_0-72h_) to changes in intrinsic solubility under fasted (solid lines) and fed (dashed lines) conditions. (JPG 1465 KB)

**Supplementary Figure 3** Sensitivity of the simulated/observed (A) maximum plasma concentration (C_max_) and (B) area under the plasma concentration-time curve from time zero to 72 hours (AUC_0-72h_) to changes in effective permeability under fasted (solid lines) and fed (dashed lines) conditions. (JPG 1413 KB)

**Supplementary Figure 4** Sensitivity of the simulated/observed (A) maximum plasma concentration (C_max_) and (B) area under the plasma concentration-time curve from time zero to 72 hours (AUC_0-72h_) to changes in the logarithm of the bile micelle-to-water partition coefficient for the neutral species (log K_m:w,neutral_; green) and the ionized species (log K_m:w,ion_; black) under fasted (solid lines) and fed (dashed lines) conditions. (JPG 1443 KB)

Supplementary file5 (DOCX 66 KB)

## Data Availability

Data are available from the corresponding author only on reasonable request due to privacy/ethical restrictions.

## Abbreviations

ADAM: Advanced dissolution, absorption and metabolism
AUC: Area under the plasma concentration–time curve
AUC_0-12h_: Area under the plasma concentration–time curve from time zero to 12 h
AUC_0-72h_: Area under the plasma concentration–time curve from time zero to 72 h
AUC_inf_: Area under the plasma concentration–time curve from time zero to infinity
B/P: Blood-to-plasma concentration ratio
BCR-ABL: Breakpoint cluster region-Abelson
CI: Confidence interval
C_max_: Maximum plasma concentration
Cp: Plasma concentration
EMA: European Medicines Agency
fa: Fraction absorbed
FaSSIF: Fasted state simulated intestinal fluid
FDA: Food and Drug Administration
FeSSIF: Fed state simulated intestinal fluid
fu_mic_: Fraction unbound in microsomes
fu_p_: Fraction unbound in plasma
GMR: Geometric mean ratio
HLM: Human liver microsomes
ISEF: Intersystem extrapolation factor
K_m_: Michaelis constant
Kp: Tissue-to-plasma partition coefficient
K_sp_: Solubility product constant
Log K_m__:w__,neutral_: Logarithm of bile micelle-to-water partition coefficient for neutral species
Log K_m__:w__,ion_: Logarithm of bile micelle-to-water partition coefficient for ion species
Log P: Logarithmic of the octanol–water partition coefficient
PBPK: Physiologically based pharmacokinetic
P_eff_: Effective permeability
pK_a_: Logarithm of acid dissociation constant
rhCYP: Recombinant cytochrome P450
S_0_: Intrinsic solubility
T_max_: Time to maximum plasma concentration
V_max_: Maximum reaction velocity
V_ss_: Volume of distribution at steady state
