# Supplementary material for: Physiologically Based Pharmacokinetic Modeling for Mechanistic Analysis of Food Effects: A Case Study with Radotinib
Source: Pharm Res. 2026 Aug 21;43(9):3159–71. doi: 10.1007/s11095-026-04179-0 (PMC13642456; doi:10.1007/s11095-026-04179-0)
Supplement: Supplementary file 1 [file 11095_2026_4179_MOESM5_ESM.docx]

**Supplementary Table**

**Table SI** Fold-changes in simulated geometric mean ratios (GMRs) relative to the baseline simulation in the joint perturbation analysis using low, baseline, and high parameter values

| **P_eff_^*^** | **Intrinsic solubility^*^** | **Log K_m:w,neutral_^**^** | **Fold-changes for AUC_0-72h_** | **Fold-changes for C_max_** |
| --- | --- | --- | --- | --- |
| Baseline | Baseline | Baseline | 1.00 | 1.00 |
| Low | Low | Low | 2.75 | 2.76 |
| Low | Low | Baseline | 2.88 | 2.83 |
| Low | Low | High | 1.71 | 1.72  2.62 |
| Low | Baseline | Low | 2.41 |  |
| Low | Baseline | Baseline | 1.67 | 1.71 |
| Low | Baseline | High | 0.48 | 0.40 |
| Low | High | Low | 1.57 | 1.89 |
| Low | High | Baseline | 0.77 | 0.75 |
| Low | High | High | 0.45 | 0.39 |
| Baseline | Low | Low | 2.75 | 2.84 |
| Baseline | Low | Baseline | 2.71 | 2.76 |
| Baseline | Low | High | 1.02 | 1.01 |
| Baseline | Baseline | Low | 2.28 | 2.55 |
| Baseline | Baseline | High | 0.37 | 0.30 |
| Baseline | High | Low | 1.00 | 1.10 |
| Baseline | High | Baseline | 0.36 | 0.29 |
| Baseline | High | High | 0.36 | 0.29 |
| High | Low | Low | 2.40 | 2.58 |
| High | Low | Baseline | 1.32 | 1.50 |
| High | Low | High | 0.30 | 0.21 |
| High | Baseline | Low | 1.06 | 1.39 |
| High | Baseline | Baseline | 0.30 | 0.21 |
| High | Baseline | High | 0.29 | 0.15 |
| High | High | Low | 0.32 | 0.23 |
| High | High | Baseline | 0.29 | 0.15 |
| High | High | High | 0.29 | 0.15 |

AUC_0-72h_, area under the plasma concentration-time curve from time zero to 72 hours; C_max_, maximum plasma concentration; Log K_m:w,neutral_, logarithmic micelle-to-water partition coefficient for neutral species; P_eff_, effective permeability

**^*^** Low and high parameter values correspond to one-tenth and 10-fold of the baseline value, respectively.

**^**^** Low and high parameter values correspond to -1 and +1 log units from baseline, respectively**.**
